# Genetic characterization of the first detected human case of low pathogenic avian influenza A/H9N2 in sub-Saharan Africa, Senegal

**DOI:** 10.1080/22221751.2020.1763858

**Published:** 2020-05-29

**Authors:** Mamadou Malado Jallow, Amary Fall, Mamadou Aliou Barry, Boly Diop, Sara Sy, Déborah Goudiaby, Malick Fall, Vincent Enouf, Mbayame Ndiaye Niang, Ndongo Dia

**Affiliations:** a Département de Virologie, Institut Pasteur de Dakar, Dakar, Sénégal; b Unité d'Epidémiologie des maladies infectieuses, Institut Pasteur de Dakar, Dakar, Sénégal; c Division surveillance épidémiologique et riposte vaccinale du ministère de la Santé et de l'action sociale, Dakar, Senegal; d Département de Biologie Animale, Faculté des Sciences et Techniques, Université Cheikh Anta DIOP de Dakar, Dakar, Sénégal; e Institut Pasteur Paris, Plateforme P2M, Dakar, Senegal

**Keywords:** Influenza, AIV, A/H9N2, phylogeny, low pathogenicity

## Abstract

The H9N2 influenza virus has become one of the dominant subtypes of influenza virus circulating in poultry, wild birds, and can occasionally cross the mammalian species barrier. Here, we report the first human A/H9N2 in Sub-Saharan Africa. The patient was a child of 16 months' old living in the South-West of Senegal. He had no influenza vaccination history and no other disease history. He had symptoms of fever with an auxiliary temperature of 39.1°C. Respiratory symptoms were an intense cough, runny nose and pulmonary crackles. All eight genome segments belonged to the A/H9N2 AIV subtype and the strain characyerized as of low pathogenicity with a RSSR/GLF amino acids mo­tif. Phylogenetic analysis of both complete HA and NA gene segments showed that the A/H9N2 subtype virus from Senegal belonged to the G1 lineage. This human case highlights the weakness of influenza surveillance in animals and the need for enhanced surveillance using a one-health approach.

In recent years, avian influenza viruses (AIVs) have been reported to intermittently infect humans. Because of the high morbidity and mortality of the infection, avian influenza viruses have become a widespread public concern. AIVs can be broadly categorized into two groups based on molecular markers in the hemagglutinin (HA) that affects their pathogenicity in chickens: the highly pathogenic avian influenza viruses (HPAIV) which display high pathogenicity in chickens and contain polybasic cleavage sites in HA, and the low pathogenicity avian influenza viruses (LPAIVs) characterized by low pathogenicity in chickens and mono- di- or occasionally tri-basic cleavage sites in haemagglutinin. So far, only the H5 and H7 subtypes have shown the HPAIV phenotype. A/H9N2, the focus of the present study, is a LPAIV subtype. The H9N2 influenza virus, isolated for the first time from turkeys in 1966 [1], is a prominent member of the influenza A family. Indeed, since its discovery, H9N2 avian influenza viruses have been detected in domestic poultry and wild birds in North America, then detected from multiple species of Europe, Africa, Asia, and the Middle East. Now, the A/H9N2 avian influenza virus is widely distributed in different regions of the world and has become one of the dominant subtypes of influenza virus circulating in poultry and wild birds [2]. In poultry, A/H9N2 infections cause a decline in egg production, with occasional high mortality [3]. A/H9N2 subtype can occasionally broaden its host range by crossing the mammalian species barrier. Indeed, A/H9N2 virus infection in pig farms has been reported in Hong Kong and China [4,5]. However, several human infections with A/H9N2 have been reported from Hong Kong and other provinces of China, exhibiting mild respiratory disease, potentially posing a threat to public health [6,7].

Another disturbing facet of the virus is that prior phylogenetic analysis showed that the influenza A/H9N2 viruses have contributed to many recent zoonotic events by providing some segments to reassortment viruses involved in zoonotic transmission [8].

The A/H9N2 LPAIV is classified into five lineages, one of which circulates in wild birds and four in poultry; two of these four lineages have previously been detected in humans [9].

A/H9N2 viruses have been isolated from several African countries; the virus appears endemic in poultry in Egypt and has been repeatedly isolated from chickens in Libya and Tunisia [10]. Additionally, since 2016 the virus has been isolated for the first time in countries across North and West Africa including Morocco, Burkina Faso, Ghana and Algeria as well as in East Africa in Uganda [10]. All viruses isolated from poultry in Africa have been of the G1 “Western” sub-lineage, related to those circulating in the Middle East area.

As of June 2019, there have been a total of 59 laboratory-confirmed human H9N2 infections with over half of those being recorded since 2015 [10]. The majority confirmed infections were young children (39 of 56 cases were aged 8 years or below), the median age of infection was 4-years-old, while the mean age was 14. Regarding Africa, only 4 human cases were reported between 2014 and 2015 from Egypt where the virus is endemic in poultry.

In Senegal, no human or animal cases of AIV infection have been reported so far, despite ongoing influenza sentinel surveillance since 1996 [11]. However, until recently, this surveillance was only focused on humans.

Here, we report the first human A/H9N2 in Sub-Saharan Africa in a country that has never experienced documented A/H9N2 infection in poultry even though unusual poultry outbreaks associated with mortalities are occassionally reported.

The patient was a child of 16 months’ old living in Ziguinchor, a region in the South-West of Senegal. He had no influenza vaccination history and no other disease history. He had symptoms of fever with an auxiliary temperature of 39.1°C. Respiratory symptoms were an intense cough, runny nose and pulmonary crackles.

Following extraction of total viral RNA, the clinical sample was tested by one-step real time reverse transcription-polymerase chain reaction (rRT-PCR), using the ABI 7500 device, according to the CDC protocol for the identification of influenza A and B viruses (courtesy of the Centers for Disease Control, Atlanta, GA). The sample was confirmed as influenza A (Ct Value of 26.3). A second real-time RT-PCR round was performed for the subtyping of influenza A viruses with primers targeting hemagglutinin genes of seasonal (H1 and H3) viruses and A(H1N1)pdm09. The sample was negative for all subtypes, and was therefore characterized as unsubtypable.

Before going through full genome sequencing, 200 μl of the swab sample were inoculated in specific-pathogen-free (SPF) embryonated chicken eggs (ECEs). After 72 h of incubation at 37°C, the allantoic fluids were collected, and screened for influenza virus by hemagglutination (HA) activity titration. HA activity was measured up to the 1/16 dilution. The presence of influenza virus was also verified by qRT-PCR for the MP gene. The isolation process was performed in a Biosafety Level 3 laboratory.

Whole genome amplification was conducted by modifying a protocol previously described by Zhou et al. [12]. After amplification, PCR products were quantified with the Qubit Fluorometer (Invitrogen life technologies). The Illumina sequencing and library construction were performed by the P2M platform at Institut Pasteur Paris. In brief, Nextera XT DNA Library Preparation kit (Illumina) was used for library construction. Finally, the pooled librairies were sequenced 150 bp paired end reads on an Illumina NextSeq 500 instrument. The fastq files were generated and demultiplexed with the bcl2fastq Conversion Software v2.20 (Illumina). The contigs obtained in Fasta format were cleaned with the GeneStudio software (GeneStudio ™ Pro, version: 2.2.0.0, 8/11/2011) and Basic Local Alignment Search (BLAST) homology search programme was used to measure sequence matching of each segment. We retrieved all eight segments of the genome with high quality sequences. The sequence alignment and phylogeny were performed using the MEGA 7.0 MUSCLE and maximum likelihood, respectively. The robustness of the ML tree was assessed by bootstrap analyses of 1,000 replicates. The evolutionary distances were derived using the Tamura 3 parameter method. Bootstrap replicates with values ≥70 are shown on the trees. All genome segments sequence of A/Senegal/0243/2019 (H9N2) has been deposited in the Global Initiative on Sharing All Influenza Data (GISAID) EpiFlu database under the accession numbers EPI1671016 to EPI1671035.

The BLAST results confirmed all eight segments as belonging to the A/H9N2 AIV subtype. The Senegalese A/H9N2 strain had low pathogenicity with a RSSR/GLF amino acids mo­tif at the cleavage site of HA (335-341 [H9 numbering]) [13]. It also harboured Q226L and I155 T mutations in the HA gene, which promote preferential binding to human-like α2-6-linked sialic acid (SA α2-6) receptors. The human-to-human transmission mutations markers T105 V and A661 T [14] were also identified in the PB2 gene as well as the mammalian host-specific mutation marker M185I [15]. However the PB2 protein also had glutamin and asparagin at position 627 and 701 respectively, which is characteristic of viruses of avian origin [13]. H274Y substitution was not detected in the NA protein, suggesting sensitivity to neuraminidase (NA) inhibitors such as oseltamivir.

The maximum likelihood phylogenetic analysis of both complete HA and NA gene segments showed that the A/H9N2 subtype virus from Senegal belonged to the G1 lineage (Figure 1), and clustered with viruses identified in North and West African chickens and wild birds between 2016 and 2018 (nucleotide similarity ranging between 97.3% and 98.3% for the HA and 97.6% and 98.5% for the NA).

**Figure 1.**
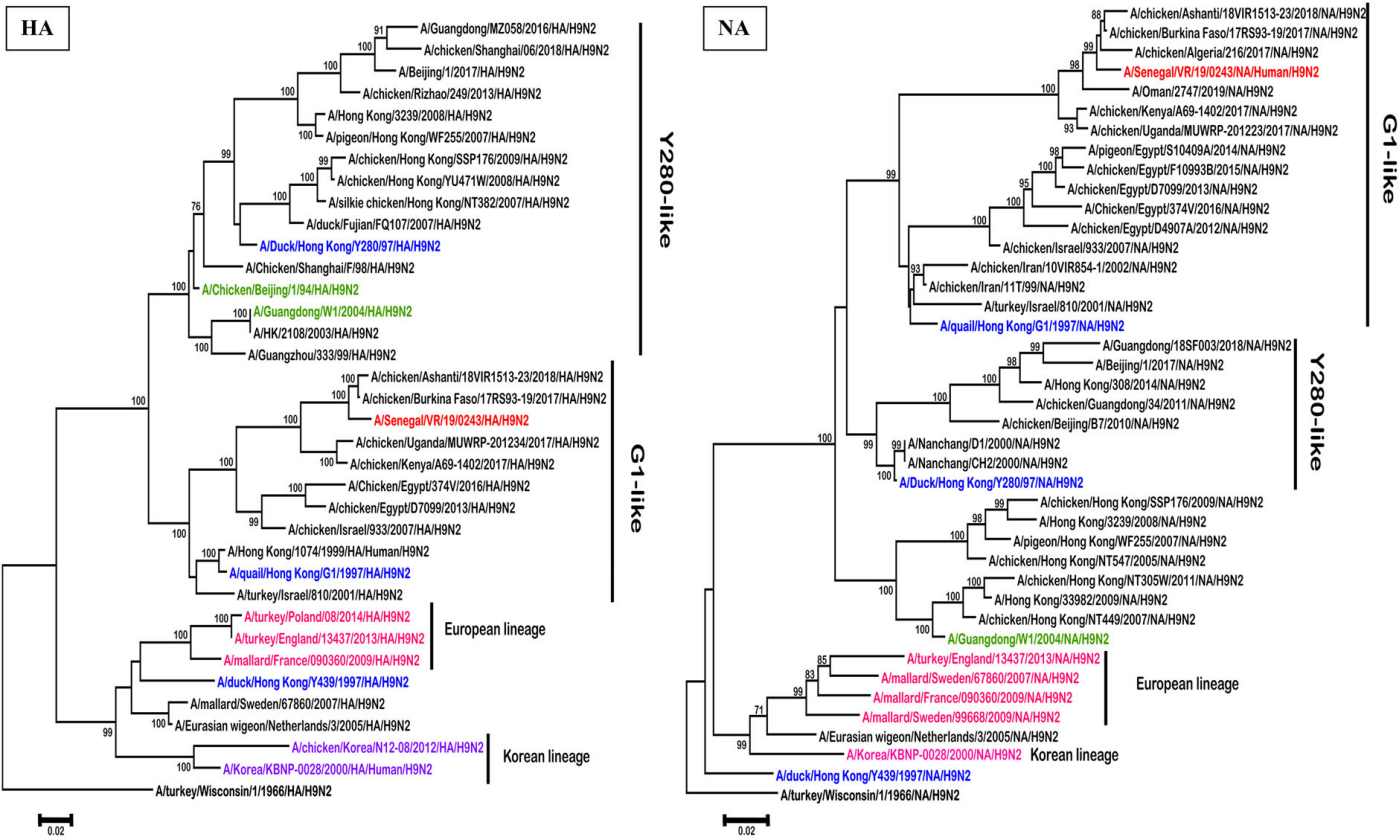
Phylogenetic analysis by maximum likelihood of the HA and NA genes of H9N2 subtype AIVs including the isolate from Senegal (in red colour)**.** The representative strains of each lineage were shown in blue and representative genotype strains were shown in different colours (green, purple, pink). Trees were generated using the Maximum Likelihood method based on the Tamura-Nei model method in MEGA 6.0. The phylogenetic trees were rooted to the old A/turkey/Wisconsin/1/1966/ isolate. We performed 1000 bootstrap replicates to determine the consensus tree; support for nodes present in >70% of the trees are displayed on branches.

Although the first human case of A/H9N2 infection was detected in 1997, the present study documents the first human case of infection in sub-Saharan Africa, despite numerous outbreaks in poultry reported in many countries (Burkina Faso, Ghana, and Uganda). Senegal has never experienced a large AIV outbreak in poultry even if unusual mortalities are regularly reported by farmers in many areas in the country. This human case highlights the weakness of influenza surveillance in animals (poultry, pigs, wild birds etc …) and the need for enhanced surveillance using a one-health approach. Therefore, monitoring markets that contain wild birds, live poultry, pigs and analyzing virus evolution and gene mutations in a timely manner are essential.
